# A Case-Based Review of Cerebral Venous Infarcts With Perfusion Imaging and Comparison to Arterial Ischemic Stroke

**DOI:** 10.3389/fradi.2021.687045

**Published:** 2021-10-14

**Authors:** Anna Y. Li, Elizabeth Tong, Vivek S. Yedavalli

**Affiliations:** ^1^Department of Radiology, Stanford University School of Medicine, Stanford, CA, United States; ^2^Department of Radiology, Johns Hopkins University School of Medicine, Baltimore, MD, United States

**Keywords:** cerebral venous infarction (CVI), cerebral venous thrombosis (CVT), MR venography (MRV), CTV, perfusion imaging

## Abstract

Cerebral venous thrombosis (CVT) and cerebral venous infarcts (CVI) are diagnostic dilemmas secondary to their rarity, non-specific symptomatology at presentation, and variable imaging features. Despite its relatively infrequence, CVT is particularly prevalent in the younger adult population and is a potentially life-threatening disease with devastating neurological complications if not addressed in a timely manner. However, when treated promptly, CVT has the potential for a more reversible course and favorable prognosis than arterial ischemic strokes (AIS). The pathophysiology of CVI is distinct from that of AIS and is closely related to its potentially reversible nature. Familiarity with the conventional and variant venous anatomy, as well as the temporal evolution of imaging findings, is crucial in establishing diagnostic confidence. The use of MR perfusion imaging (MRP) and arterial spin-labeling (ASL) can potentially aid in the diagnosis of CVT/CVI *via* characterization of cerebral blood flow. The presence and extent of a cerebral perfusion deficit on either CT or MRI may play a role in clinical outcomes for patients with CVT, although future larger studies must be performed. This review presents a case-based overview focusing on the classic imaging characteristics of CVT and CVI in conjunction with bolus MRP and ASL findings in the adult population.

## Introduction

Cerebral venous infarct (CVI) is an uncommon cause of all strokes (0.5–2%) that disproportionately affects young adults (1, 2). CVI is most commonly caused by cerebral venous thrombosis (CVT). CVT refers to the complete or partial occlusion of the dural venous sinuses and/or cerebral veins with potentially devastating clinical consequences if untreated. The diagnosis of CVT and CVI remains challenging in light of the variability in its clinical and imaging features, non-specific symptomatology, and infrequent presentation. Although CVT and CVI have an estimated mortality of 5–30%, expeditious diagnosis and treatment generally leads to a favorable prognosis with up to 80% of patients recovering without functional disability (3, 4). The incidence of CVI has been estimated at 1.32–1.57 per 100,000 person-years, 13.2 cases per million per year, 2–7 million people annually, or 5–8 cases encountered annually at a typical tertiary care center (4–6). Approximately 50% of cerebral venous occlusions progress to CVI, which constitute rare, non-arterial distribution infarcts secondary to increases in venous pressure resulting in ischemia (6–8). For this reason, a recent study has also defined CVI as venous parenchymal lesions with three subtypes, namely, hemorrhagic ischemia (HI), intracerebral hematoma (ICH), and non-hemorrhagic ischemia (NHI) (9). For the purposes of this review, we refer to these lesions as CVI. The potential for reversibility of the disease process along with the morbidity of its acute complications and long-term sequelae motivates the early diagnosis and management of CVT and CVI.

The clinical features, pathophysiology, diagnosis, and management of CVT and CVI are well-documented in the current literature. Ahmed et al. explored the normal cerebral venous anatomy and assessed the frequency of its anatomic variants (10). Saposnik et al. presented a comprehensive scientific statement from the American Heart Association/American Stroke Association with an algorithm for the diagnosis of CVT accompanied by tiered evidence-based management recommendations (1). Schaller and Graf provided insight into the pathophysiology of CVT by exploring patterns of parenchymal injury from animal model studies of cerebral venous occlusion (8). The natural history and prognosis of CVT was detailed in the International Study on Cerebral Vein and Dural Sinus Thrombosis (ISCVT), which found that the prognosis of CVT is improving overall, and that coma, cerebral hemorrhage, and malignancy are prognostic factors for death or dependence (11). Another group found that the most recent patterns of parenchymal abnormalities in 44 consecutive patients hospitalized with CVT were HI (56.8%), ICH (22.72%), and NHI (20.45%) (9). Multiple other studies have addressed the anatomic distribution of CVI by frequency (1, 2, 4, 5, 12). Although most cases of sinus thrombosis involve multiple sinuses, the superior sagittal sinus (SSS) is the most common site of dural sinus thrombosis followed by the transverse sinus (TS) and sigmoid sinus (SS) (1, 4, 5). The deep cerebral venous system ranks behind the dural sinuses in frequency with the internal cerebral veins (ICV), the vein of Galen (VG), the straight sinus (StS), and the jugular bulb (JB) in order of descending frequency of venous thrombosis (5). Superficial/cortical venous thrombosis, particularly when isolated, was the most uncommon site of CVT (5). SSS thrombosis is often associated with concurrent cortical vein thrombosis (12).

The wide range of normal variant anatomy and collateralization in the cerebral venous system both influence and may confound the imaging diagnosis of CVT and CVI. Therefore, the purpose of this case review is to highlight CVI as a rare non-arterial form of stroke, which is an often-underdiagnosed entity in the adult population. In this overview, we outline the anatomy and pathophysiology of CVT and CVI and present a series of cases depicting the classic imaging findings of CVT and CVI using the most up-to-date diagnostic imaging techniques. We also emphasize the imaging appearance of CVT and CVI by anatomic location and chronicity of findings on computed tomography (CT) and magnetic resonance imaging (MRI). In particular, we provide updates to the current body of literature regarding the imaging appearance of CVT and CVI on MRI in conjunction with a case-based approach that highlights the imaging findings on perfusion sequences. To the best of our knowledge, there are no dedicated studies to date that explore perfusion with arterial spin-labeling (ASL) in CVI and with both bolus dynamic susceptibility contrast MR perfusion imaging (MRP) and ASL imaging in CVT and CVI. We hope that this overview heightens the reader's familiarity with CVT and CVI on conventional and perfusion imaging for daily clinical practice.

## Epidemiology

**Figure 1:**
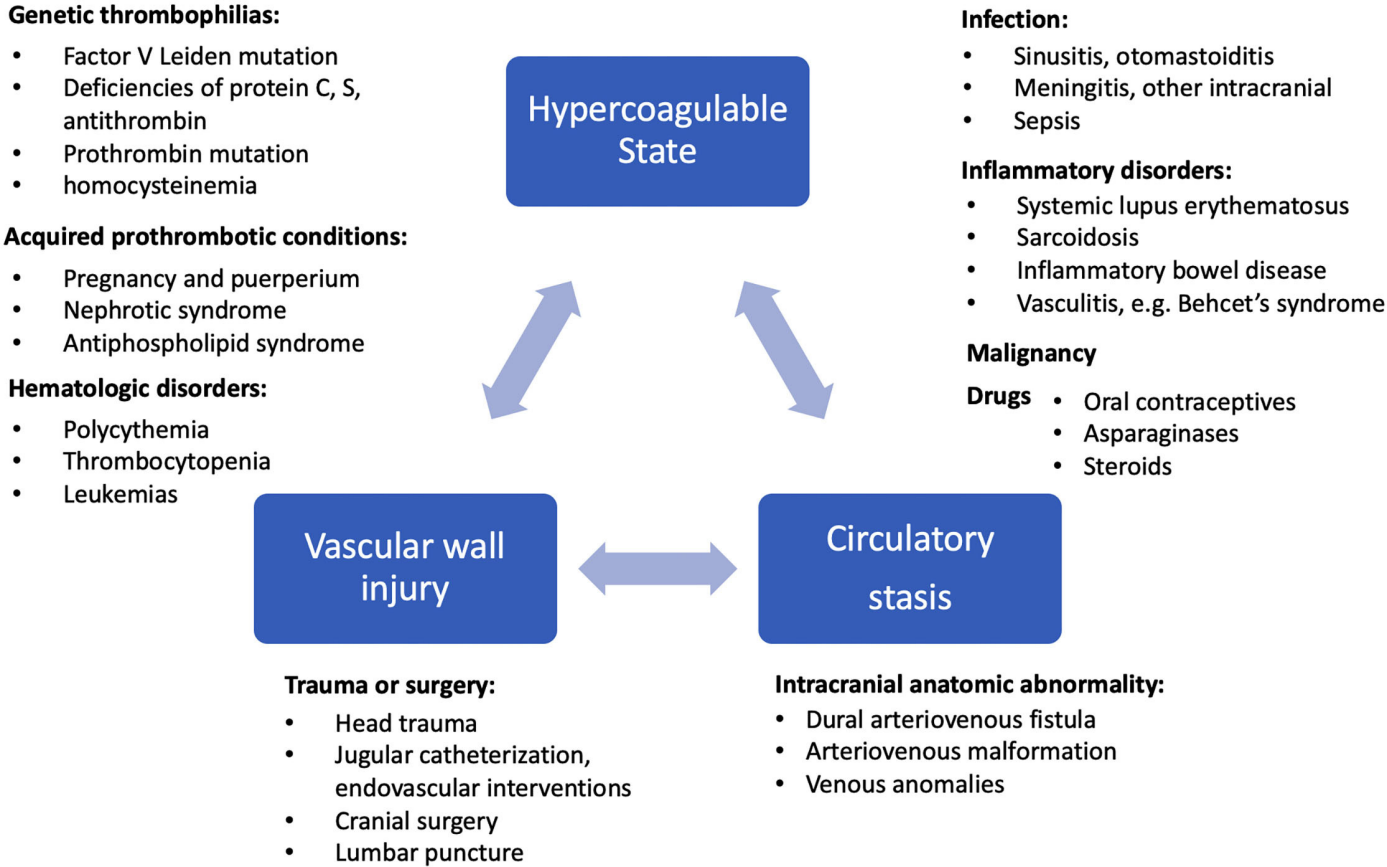
Etiology and risk factors of CVI are shown in relation to Virchow's triad of hypercoagulable state, circulatory stasis, and vascular wall injury (13, 16).

While arterial ischemic strokes (AIS) are overwhelmingly seen in the older population, CVT/CVI is much more prevalent in young adults (particularly under 50 years of age and among women of childbearing age), affecting up to 3.7–5.3 times more female than male patients with minimal sex differences in age groups over 60 years (1, 4, 13). However, CVI in the postoperative neurologic surgery setting is more common in the elderly population (14). Whereas, AIS are predominantly associated with risk factors of cardiovascular disease such as atherosclerosis, diabetes, hypertension, and atrial fibrillation leading to ischemia, the etiologies of CVT can be characterized by Virchow's triad of hemodynamic stasis, endothelial injury, and hypercoagulable states (15). As presented in Figure 1, the risk factors of CVI can be explained by genetic prothrombotic conditions, as well as acquired risks. Genetic prothrombotic conditions include inherited thrombophilias (e.g., factor V Leiden and also deficiencies of protein C, protein S, and antithrombin III) (2, 4). Acquired risks include trauma, surgery, sepsis, nephrotic syndrome, pregnancy and the puerperium, antiphospholipid syndrome, malignancy, exogenous hormones, and other inflammatory disease states (e.g., inflammatory bowel disease, nephrotic syndrome, Behcet's disease) (1, 4). Between 20 and 35% of CVT patients were found to have an inherited or acquired prothrombotic condition (7).

## Anatomy

Unlike the systemic veins, the cerebral veins and sinuses are valveless and, consequentially, have bidirectional flow (7). Additionally, the drainage of the cerebral venous system does not mirror that of the cerebral arterial distribution as is the case with the systemic circulation, leading to distinctive patterns of parenchymal lesions in CVI (7, 15). The intracranial venous system can be divided into the dural venous sinuses and cerebral veins, which are further partitioned into the superficial and deep cerebral veins. The dural venous sinuses may be subdivided into an anteroinferior group and a more prominent posterosuperior group. The posterosuperior group consists of the SSS, inferior sagittal sinus (ISS), StS, sinus confluence or torcula herophili, TS, SS, and JB (5, 7). The SSS drains most of the blood from the surface of the cerebral hemispheres *via* superficial cortical veins, the anastomotic vein of Trolard (VT), and receives drainage from the emissary, meningeal, and calvarial diploic veins (7, 10). The less prominent anteroinferior group consists of the cavernous sinus (CS), superior and inferior petrosal sinuses, clival venous plexus, and sphenoparietal sinus (7). The superficial cerebral veins course over the cerebral convexity and drain perpendicularly into the SSS. The three main anastomotic superficial cortical veins include the superficial middle cerebral vein, the VT, and the vein of Labbé (VL). The VT receives drainage from the superficial middle cerebral vein and drains into the SSS, whereas the VL courses over the inferolateral temporal lobe and drains into the TS (2, 7). The deep cerebral veins consist of medullary and subependymal veins that drain into the ICV, VG, and StS toward the sinus confluence (2, 7).

**Figure 2:**
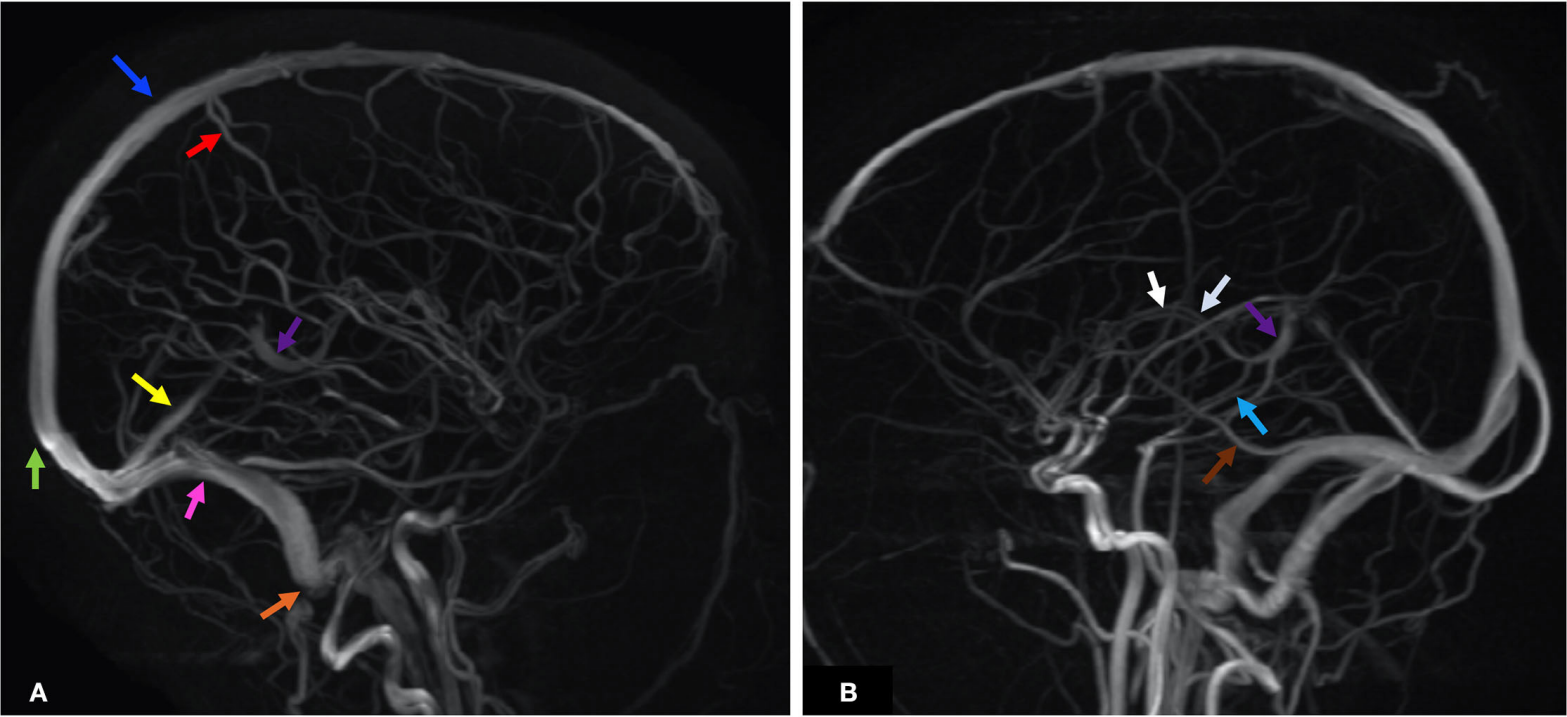
MRV maximum intensity projection (MIPS) of two patients illustrates the anatomy of the dural venous sinuses and prominent cerebral veins. Each anatomic structure is labeled by colored arrows as below. **(A)** Blue—SSS, green—SC, red—VT, yellow—StS, purple—VG, pink—TS, orange—SS. **(B)** White—ICV, purple—VG, light blue—VR, brown—VL. ICV, internal cerebral veins; SC, sinus confluence (torcular herophili); SS, sigmoid sinus; SSS, superior sagittal sinus; StS, straight sinus; TS, transverse sinus; VG, vein of Galen; VL, vein of Labbé; VT, vein of Trolard; VR, basal vein of Rosenthal.

**Figure 3:**
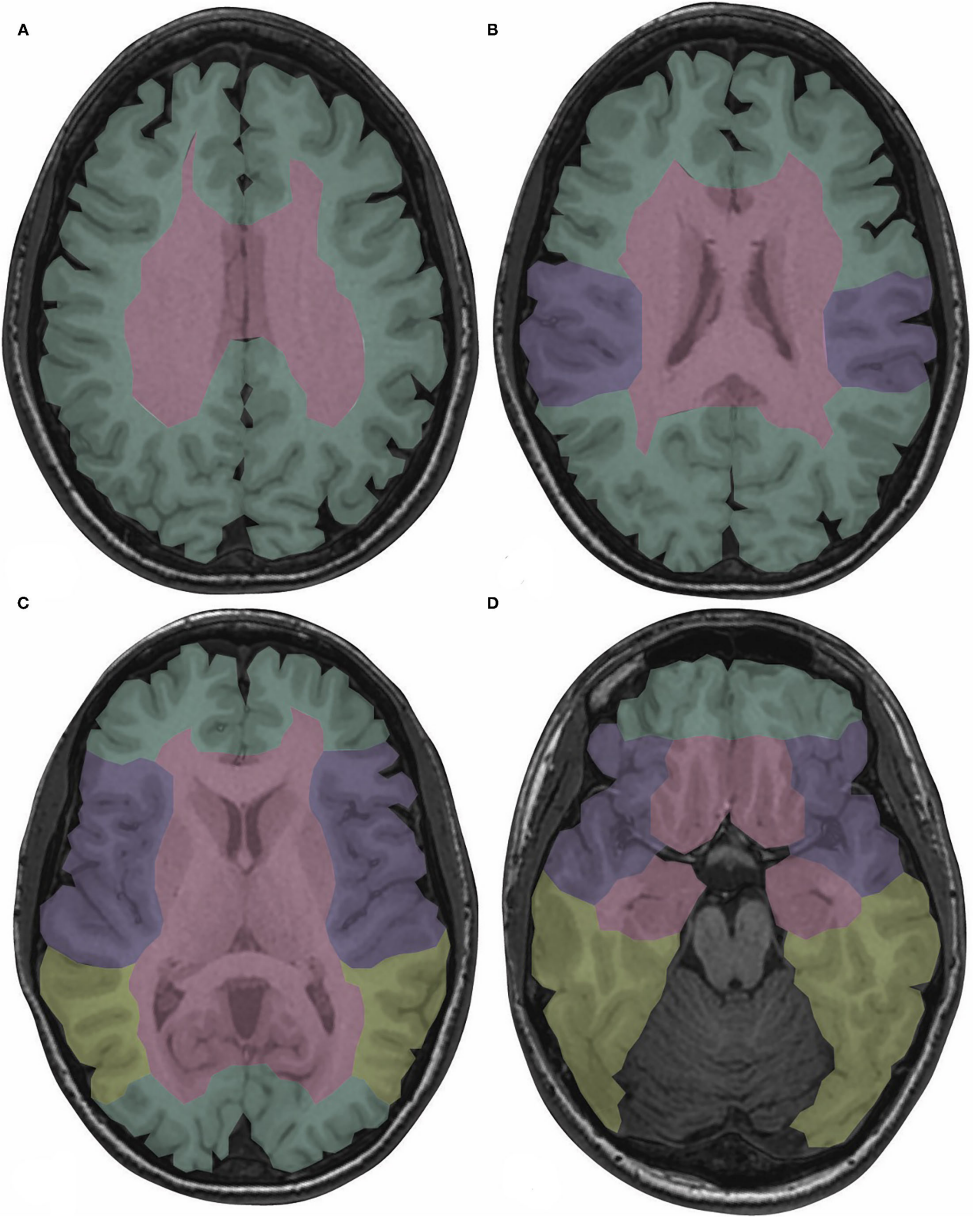
Drainage territories of the cerebral venous system are color-coded on T1-weighted images depicting normal brain anatomy at the levels of the centrum semiovale **(A)**, body of the lateral ventricles **(B)**, basal ganglia **(C)**, and midbrain **(D)**. The superficial/cortical veins drain the cortex, subcortical white matter, and SSS (green). The deep cerebral veins drain the basal ganglia, thalami, internal capsules, and third and lateral ventricles (red). The sphenoparietal and cavernous sinuses drain the peri-Sylvian region (purple). The TS and VL, if dominant, drain the posterior temporal and inferior parietal lobes (yellow) (5, 7). SSS, superior sagittal sinus; TS, transverse sinus; VL, vein of Labbé.

The CS drains venous blood from the orbits and the inferior frontal and parietal lobes (10). Non-jugular venous pathways include the vertebral plexus and pterygopalatine plexus and may play a role in collateral drainage in the setting of CVT and resultant intracranial hypertension (7). Figure 2 presents the normal dural venous sinus and cerebral venous anatomy as seen on MR venography (MRV). Figure 3 illustrates the drainage patterns of the cerebral venous system including the superficial/cortical, deep, inferolateral, and posterolateral patterns, which is more variable than those of the cerebral arterial system.

### Normal Variant Anatomy

Asymmetry of the TS is among the most common of the dural venous sinus anatomic variants (10). The right TS is often more prominent than the left; a hypoplastic and stenotic left TS is seen in up to 39% of the population (10). The SSS may have a hypoplastic or absent anterior segment. An asymmetric or high bifurcation of the torcula herophili may mimic the empty delta sign seen on contrast-enhanced CT (CECT) or MRI (5, 7). Prominent arachnoid granulations and fibrous septations, classically within the TS, may mimic filling defects on CT venography (CTV) or MRV (5). A hypoplastic TS or SS may be seen concomitantly with alternative venous drainage pathways such as a persistent occipital sinus or prominent mastoid emissary veins (5, 7). The three named superficial anastomotic veins (superficial middle cerebral vein, VT, and VL) vary greatly in size and have a reciprocal relationship such that the non-dominant third anastomotic vein is often aplastic or hypoplastic (7). Expected lack of signal or flow on CT or MRI within a normal variant hypoplastic sinus may mimic abnormal absent flow in the setting of venous sinus thrombosis.

## Pathophysiology of CVT/CVI

Local effects related to venous congestion drive the pathophysiology of cortical and deep cerebral vein thrombosis (8, 16). Intraluminal or extrinsic cerebral venous occlusion leads to dilation of the venous and capillary bed, resulting in elevated venous pressure and reduced capillary perfusion pressure typically with an increase in cerebral blood volume (CBV) and interstitial brain edema (8, 17, 18). Blood–brain barrier (BBB) disruption occurs secondary to erythrocyte diapedesis in the setting of increased venous and capillary pressure and results in vasogenic edema (3, 5, 8, 15). If the rise in venous pressure is rapid and in the absence of adequate venous collateral recruitment, the valveless and friable cerebral veins may be unable to withstand the pressure gradient, leading to rupture of the cerebral veins and parenchymal hemorrhage (6, 8). In the absence of adequate compensatory venous collaterals and prior to thrombus recanalization, rising venous pressure results in reduction of arterial perfusion pressure; following diminished cerebral blood flow (CBF), disruption of the Na+/K+ ATPase pump ensues with the eventual endpoint of cell death, cytotoxic edema, and potential infarction (5). In the setting of dural sinus thrombosis, increased venous sinus congestion leads to impaired cerebrospinal fluid (CSF) reabsorption with resultant intracranial hypertension (8, 16).

## Clinical Features of CVT/CVI

The diverse and non-specific clinical presentations of CVT/CVI make this entity a particularly challenging diagnosis. Approximately 80% of patients with CVT present in the acute to subacute phase and 20% in the chronic phase, with a median time from onset to diagnosis of ~7 days (4, 16). In the acute phase of the disorder, cerebral herniation caused by cerebral edema is the most common complication to result in death and is seen in ~5–13% of cases (1, 2, 4, 16). After an episode of CVT, the overall risk of recurrence of any thrombotic event is ~6.5% (1).

Clinical findings may be related to increased intracranial pressure in the setting of impaired venous drainage or focal parenchymal injury secondary to venous ischemia, infarction, or hemorrhage (1). Headache, suggestive of increased intracranial pressure, is the most frequent but least specific symptom at presentation (16). Seen in up to 90% of CVT patients, headaches may be diffuse with progressive worsening in severity over days to weeks and may be diffuse or present with unilateral or focal symptoms in up to 67% of cases (1, 4, 13, 16). Thunderclap and migraine-type headaches have both been described with thunderclap headaches suggestive of subarachnoid hemorrhage (1, 2). Isolated headaches without other symptoms such as focal neurologic deficits or papilledema are seen in up to 25% of patients with CVT (1). Occipital or neck pain may be more prevalent in SS thrombosis or ipsilateral SS and TS thrombosis (4). Absence of headache in up to 10% of cases may be seen more commonly with men, the elderly, cancer patients, and isolated cortical vein thrombosis (3). Seizures are found in up to ~40% of patients with CVT, with generalized seizures more common than focal seizures (3, 4). Predictors of acute seizures in CVT include focal parenchymal injury, hemorrhagic infarction, frontal lobe involvement, SSS and cortical vein thrombosis, high D-dimer levels, and altered consciousness with a Glasgow Coma Scale (GCS) score of <8 (11, 19). Status epilepticus has been observed in up to 5–7% of patients with CVI (4). Encountered in one-third to one-half of CVT cases, focal neurologic deficits include the spectrum of motor and sensory impairment, hemiplegia, aphasia, cranial nerve (CN) palsies, and cortical blindness; among these, motor deficits were the most prevalent (seen in 19–39% of CVT patients) and have been particularly associated with SSS, cortical vein, or deep cerebral vein occlusion (2, 4, 13). Approximately 12% of CVT patients demonstrate CN involvement that may include CN III–X and XII (4). Ophthalmologic symptoms such as papilledema and constriction or loss of the visual field are relatively common. More common in patients with cortical hemorrhage or chronic CVT, papilledema is seen in up to 68% of patients and is rare without concomitant headache (4). Seen in up to 13% of CVT cases, acute vision impairment may manifest as bilateral homonymous hemianopsia or total blindness and is often reversible with treatment; however, progression to cortical blindness may occur if the geniculocalcarine tract and the primary visual cortex are sites of infarction or hemorrhage (4). Chronic vision loss may result from secondary optic atrophy in the setting of long-term papilledema (4, 13, 16). Altered consciousness, cognitive impairment, amnesia, and coma are seen with deep venous system and StS CVI, whereas encephalopathy and disorientation are seen with VL thrombosis (7, 20). Neuropsychiatric manifestations, such as amnesia and confusion, as well as psychosis, are also associated with deep venous thrombosis (4). Several cohort studies have noted clinical features associated with poor prognosis in CVT to be coma, encephalopathy, decreased consciousness, hemiparesis, intracranial hemorrhage, and seizures in addition to demographic factors of male sex and age > 37 years (1, 4). Table 1 summarizes the clinical presentations of CVT by frequency, and Table 2 presents the particular clinical presentations associated with specific anatomical locations of CVI. Early complications of CVT include: (1) hydrocephalus, more frequently communicating hydrocephalus secondary to impaired CSF absorption *via* the arachnoid granulations and rarely obstructing hydrocephalus from intraventricular hemorrhage; (2) intracranial hypertension, which may be isolated in up to 40% of patients with CVT; and (3) seizures (1, 2, 16). Late complications noted upon follow-up of CVT consist of: (1) residual headache, (2) visual loss, particularly with clinical presentations of increased intracranial pressure and in the setting of delayed diagnosis, (3) seizures, and (4) dural arteriovenous fistulas (1).

**Table 1:** An overview of the clinical presentations of CVT/CVI by frequency (1, 4, 13, 16, 20).

**Clinical feature**	**Frequency at presentation**
Headache	80–90%
**Ophthalmologic symptoms**	
Papilledema	28–68%
Vision impairment	13%
Seizure	32–39%
Focal neurologic deficits	50%
Motor impairment	19–39%
Aphasia	19–24%
Cranial nerve palsy	12%
Altered level of consciousness	20–30%

**Table 2:** Summary of distinctive clinical features of CVT/CVI that can manifest specific to the anatomic location of thrombosis (1, 2, 13, 16).

**Location**	**Clinical features**
SSS	Headache
TS	Earache, mastoid region pain, CN 7-8 palsy
Left TS*	Aphasia (fluent)
SS	CN 7-8 palsy
iPS	CN 6 palsy
CS	Conjunctival swelling, exophthalmos, oculomotor nerve damage, CN 3-6 palsy
CV	Aphasia, limb paresis, seizures
StS	Coma, encephalopathy, amnesia, delirium, mutism
VL	Aphasia, contralateral hemiparesis

*SSS, superior sagittal sinus; TS, transverse sinus; SS, sigmoid sinus; StS, straight sinus; CS, cavernous sinus; CV, cortical vein; VL, vein of Labbé; iPS, inferior petrosal sinus; CN, cranial nerve*.

## Treatment of CVI

CVT may be provoked or unprovoked by existing risk factors, a distinction which influences management. Despite concomitant intracranial hemorrhage, several randomized clinical trials demonstrated significant mortality benefit with heparin [low-molecular-weight heparin (LMWH) and unfractionated heparin (UFH)] in the management of CVT (4). Prompt initiation of anticoagulation limits thrombus growth, promotes recanalization, and prevents deep venous thrombosis and pulmonary embolism (1). When anticoagulation alone is inadequate, such as with extensive thrombus burden or in settings of clinical deterioration during heparin treatment, more invasive approaches may be applied including direct catheter chemical thrombolysis or mechanical thrombectomy with or without thrombolysis (2, 13, 16). As seizures are more likely to occur when parenchymal lesions accompany CVT, early initiation of antiepileptics is recommended (1). In the circumstance of postoperative venous injury, cerebral venous reconstruction such as *via* saphenous vein grafts or anastomosis with an adjacent cortical vein may improve the morbidity (14). With impending cerebral herniation due to severe mass effect from edema and/or hemorrhage, emergent decompressive craniectomy may be a lifesaving measure similar to its indications in AIS (1).

## Comparison of CVT/CVI vs. AIS

### Epidemiology of CVI vs. AIS

Distinguishing CVI from AIS is clinically important as their treatment and prognosis differ. While AIS is more frequently encountered in the elderly population, CVI is overwhelmingly seen in the young adult population, particularly in women of child-bearing age given the female-related risk factors such as pregnancy, the postpartum state, and the use of oral contraceptives. The risk factors of CVI and AIS reflect their propensity for different populations. CVI have a much greater association with inflammatory and hypercoagulable states, as well as infection, particularly in the setting of CS thrombosis, whereas AIS most commonly affect those with atherosclerotic disease risk factors. AIS can arise from occlusion of an artery with subsequent ischemia of the downstream parenchymal distribution, with two-thirds of cases caused by thrombi and one-third by emboli, resulting in imaging findings within classic arterial distributions (15).

### Clinical Presentation of CVI vs. AIS

While the classic clinical presentation of patients with AIS is hemiparesis or other focal neurologic deficits, the clinical presentation of CVI is more variable and non-specific with persistent and progressively worsening headache as the predominant symptom among seizures, ophthalmologic symptoms such as papilledema, encephalopathy, and even psychosis (4, 13). Deep CVT more often presents with decreased consciousness, coma, rapid neurologic deterioration, encephalopathy, amnesia, and lack of focal neurologic findings, many of which are presentations associated with poor clinical prognosis (1, 13).

### Treatment of CVI vs. AIS

The treatment of AIS is dependent upon timely initiation of intravenous thrombolysis with tissue plasminogen activator (tPA) when appropriate, intra-arterial thrombolysis, and thrombectomy, as well as oral anticoagulation and antiplatelet medication. Despite the more frequent occurrence of parenchymal hemorrhage in CVI than in AIS, antithrombotic therapy with heparin and oral anticoagulants have shown mortality benefit in randomized controlled trials (RCTs) of LMWH and UFH with intravascular chemical and/or mechanical thrombolysis as an option when patients exhibit continued deterioration despite the initiation of anticoagulation (1, 4, 13).

### Prognosis and Mortality of CVI vs. AIS

Several important factors likely explain the improved neurologic outcomes with CVI as compared with AIS. Cerebral venous occlusion results in functional and metabolic derangements at the tissue level with less irreversible damage, particularly at the neuronal level, than cerebral arterial occlusion (8). Despite elevated venous pressure and congestion in the setting of CVT, compensation *via* venous dilation and recruitment of venous collaterals may still allow for adequate brain tissue perfusion at lower flow rates, at least early in the progression of venous occlusion (6, 8, 15). Vasogenic edema and cytotoxic edema both play a role in CVT, whereas cytotoxic edema features more prominently in the pathophysiology of arterial strokes (8).

Parenchymal hemorrhage is often noted as a more common finding with venous rather than arterial infarcts; however, their etiologies vary. Increased venous and capillary pressure and rupture of cerebral veins account for parenchymal hemorrhage in the setting of CVT, whereas hemorrhagic conversion of arterial infarcts is caused by reperfusion of ischemic brain tissue (8, 15, 18). Table 3 summarizes the defining pathophysiologic and imaging characteristics that distinguish arterial vs. venous etiology of strokes.

**Table 3:** Summary of the defining characteristics of cerebral arterial ischemic vs. venous infarcts (AIS vs. CVI) with highlights on the distinctive imaging features of each entity in relation to the pathophysiology (1, 2, 6, 8, 11, 15).

**Characteristic**	**AIS**	**CVI**
Pathophysiology	Arterial occlusion/stenosis leads to cytotoxic edema and ischemia	Venous congestion leads to vasogenic edema and may progress to cytotoxic edema with decreased CBF
Main imaging findings	Wedge-shaped loss of gray-white differentiation secondary to cytotoxic edema	Subcortical and periventricular white matter edema with cortical sparing (mainly vasogenic) often in a rounded area, parenchymal hemorrhages (petechial vs. hemorrhagic infarct)
NECT	Dense vessel sign, loss of gray-white differentiation in an arterial distribution	Cord sign and hyperdense veins, vasogenic and cytotoxic edema in a non-arterial distribution, bilateral basal ganglia and thalamic lesions
CECT	Arterial filling defect/occlusion	Empty delta sign (venous sinus filling defect), collateral medullary veins
MRI	Edema in arterial distribution	Edema in non-arterial distribution
	GRE±	GRE+
	DWI+	DWI ± (DWI+ may correlate with decreased likelihood of recanalization)
	Decreased ASL in infarcted areas	Decreased ASL in infarcts, increased ASL may be seen proximal to thrombus within a dural sinus
CTA/MRA or CTV/MRV	Arterial occlusion or filling defect	Venous occlusion or filling detect
Perfusion	Increased MTT, decreased CBF, decreased CBV (arterial occlusion)	Increased MTT, decreased CBF, normal to increased CBV (venous congestion)
Evolution of ischemia	Less reversible	More reversible

About half of patients with AIS suffer permanent neurologic deficits compared with only 6–13% with permanent neurologic deficits after CVI (1, 15). AIS is the third most common cause of death in the United States followed by cancer and myocardial infarction, whereas CVI has an estimated mortality of 5–30% (3, 15), and deep CVT claims a mortality rate of 25% (7).

## Classic Imaging Features of CVT and CVI

CVT may be detected on CT, MRI, or venographic imaging techniques including CTV, unenhanced time-of-flight MRV (TOF MRV), and contrast-enhanced MRV (C-MRV) (5). Compared with MRV, CTV is less affected by flow-rated artifacts. The heightened sensitivity to slow venous flow makes TOF MRV an excellent imaging technique in the detection of CVT; however, venous flow in the plane of acquisition may result in signal loss due to the saturation effect, which can be ameliorated with C-MRV (2, 5).

### Imaging Appearance of CVT by Thrombus Location

The imaging appearance of CVT varies based on the anatomic location of venous occlusion with thrombosis of the dural venous sinuses, superficial/cortical cerebral veins, and deep cerebral veins all possessing overlapping, as well as distinctive imaging findings. On unenhanced CT (NECT), the hyperattenuating vein or “cord sign” refers to the hyperdense appearance of a thrombosed vein and has a reported sensitivity ranging 25–64% (2, 5, 7). The thrombosed dural venous sinuses may appear expanded and hyperdense; adjacent sulcal effacement and parenchymal vasogenic edema with or without petechial hemorrhage in the drainage territory of the thrombosed sinus may provide indirect evidence of CVT (7). On CECT and CTV, as well as MRV, the “empty delta” sign represents intensely enhancing dura surrounding an intrasinus filling defect caused by thrombus and may be seen in up to 70% of CVT cases (5, 6). Enlarged, irregular veins adjacent to the thrombosed dural sinus suggest the development of collateral venous drainage (7). The NECT may be normal in up to 25–30% of cases with CVT and does not exclude CVT when negative (7). A hyperdense dural sinus may be easily overlooked with beam-hardening artifact adjacent to the calvarium and partial-volume artifacts on axial NECT slices (2, 5).

Although a hyperdense sinus or intrasinus filling defect is a more classic finding, gyral swelling is the earliest imaging sign of CVT (6). On MRI, vasogenic edema may be seen in the form of T2 or T2 FLAIR hyperintensity of the subcortical and periventricular white matter with sparing of the overlying cortex (6). As opposed to AIS, gray-white differentiation is relatively preserved in CVI (6). Parenchymal edema without other signal abnormality or focal lesion may occur in up to 42% of patients with CVT and may be accompanied by sulcal effacement, decreased visibility of the cisterns, and reduced ventricular size (5). In a study of parenchymal lesions related to CVT in 44 consecutive CVT patients, Arnoux et al. found that SSS occlusion was seen with parietal and frontal parenchymal abnormalities, TS occlusion with inferior temporal and cerebellar parenchymal lesions, and StS occlusion with bilateral thalamic lesions (9). Vasogenic edema can be distinguished from cytotoxic edema on diffusion-weighted imaging (DWI). DWI signal abnormality with corresponding apparent diffusion coefficient (ADC) hypointensity represents cytotoxic edema and infarction, whereas T2 shine-through on ADC instead represents vasogenic edema and potential reversibility of disease (6). Parenchymal hemorrhage may be seen with cytotoxic and vasogenic edema in the setting of CVT (5). Juxtacortical hemorrhages are small hemorrhages with none to minimal surrounding edema seen almost exclusively in SSS occlusion and are specific for CVT (3). Parenchymal enhancement, seen in up to 29% of cases, is an indication of BBB disruption and can be seen with vasogenic or cytotoxic edema and thus reversible or irreversible lesions (5). The enhancement pattern is typically gyral and may be accompanied by adjacent leptomeningeal, tentorial, and cortical venous enhancement secondary to venous congestion and/or collaterals (5, 6). Flow-related effects on MRI signal intensity complicate the diagnosis as slow venous flow and flow-related enhancement may lead to a hyperdense sinus appearance (2). Susceptibility within thrombosed veins manifests as hypointense signal in a “blooming” appearance on T2^*^ gradient echo (GRE) sequences and susceptibility weighted imaging (SWI) in acute CVT with gradual resolution over time (7).

Superficial/cortical vein thrombosis appears as hyperdense cortical vessels on NECT, or the “cord sign.” Isolated cortical vein thrombosis has been considered rare (up to 5%) (7). In a retrospective analysis of 49 patients, Jang et al. noted cortical veins as the most common site of CVT at 63%, followed by SSS at 61%, which may relate to SSS thrombosis as a frequent concomitant finding along with cortical vein thrombosis (7, 12). SSS occlusion with extension to the cortical veins may present with edema and petechial hemorrhage with involvement of the cortex and subcortical white matter, as well as subarachnoid hemorrhage along the convexity (7). CVT in a dominant anastomotic vein may appear as a lobar pattern of parenchymal hemorrhage within its drainage territory; for instance, posterior temporal and anterior parietal hemorrhage is associated with thrombosis in the VL (1, 7).

The deep cerebral venous system drains the bilateral basal ganglia, thalami, and deep white matter. On NECT, dense appearance of the ICV or StS may look similar to a CECT (7). Parenchymal findings of edema and hemorrhage are generally bilateral and may result in infarction of the basal ganglia and thalami. Venographic studies may show lack of opacification in the deep venous drainage system.

Since the CS receives venous drainage from the orbits, face, and neck, CS thrombosis has a much stronger association with infections of the face in addition to otomastoiditis, odontogenic disease, trauma, and neoplasm (7). On CT, periorbital edema and fat stranding, proptosis, and lateral bulging of the CS walls, and occasionally a thrombosed superior ophthalmic vein, can be seen on NECT (5, 7). CECT findings include irregular filling defects in an expanded CS, whereas MR demonstrates non-enhancing filling defects in the CS and orbital vein thrombosis on contrast-enhanced T1-weighted images (T1 + C) (5, 7).

### Imaging Findings in the Temporal Evolution of CVT

Predictable changes in MRI signal abnormality with the stages of thrombus evolution form the basis for characterizing the chronicity of CVT. In the acute stage of CVT (days 1–5), T1 isointense and strongly T2 hypointense clot, relative to brain parenchyma on T2-weighted and fluid-attenuated inversion recovery (FLAIR) images, accompanied by absence of flow voids can be seen in the thrombosed vein or sinus, which reflects the desaturation of hemoglobin to deoxyhemoglobin within intact red blood cells (2, 5, 7). An acutely thrombosed sinus may also exhibit an expanded appearance with abnormally convex margins (7). T2 and FLAIR images may demonstrate nearby gyral edema and parenchymal hyperintensity secondary to obstructed venous outflow (7). Among all sequences, C-MRV has the highest sensitivity for visualization of acute CVT (7).

**Figure 4:**
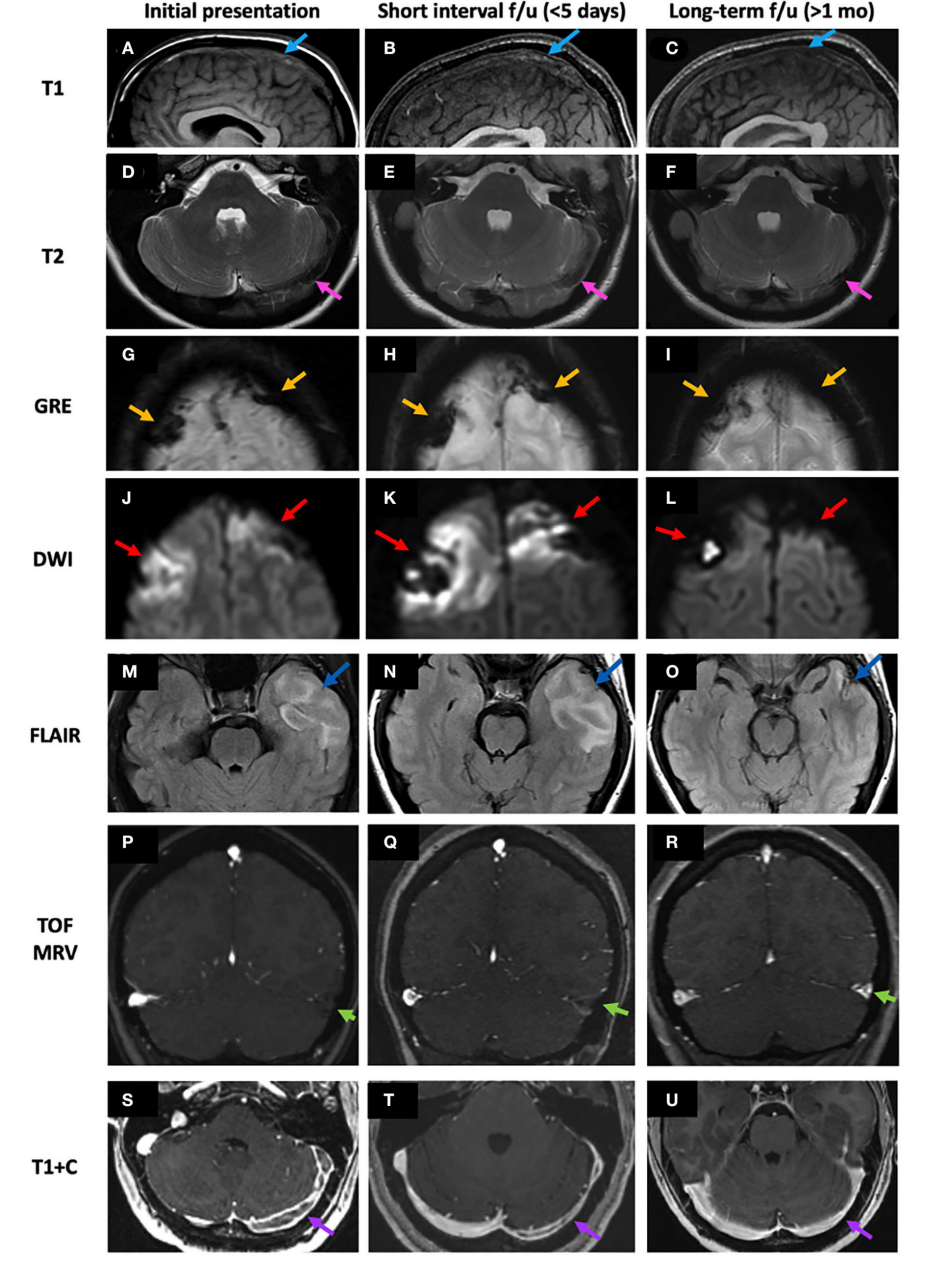
Temporal evolution of CVT on MRI from the acute to late acute to chronic phases. Intrinsic T1 signal within the SSS thrombosis (case 1) progresses from T1 isointense in the acute phase to mixed isointense/mildly hyperintense and to isointense again in the chronic phase (**A–C**, blue arrows). At the left TS thrombosis (case 3), T2 signal progresses from very hypointense to more isointense and remains mostly isointense (**D–F**, pink arrows). Bilateral frontal hemorrhagic CVI were seen in a case of SSS thrombosis (case 2). Over time, the bifrontal GRE hypointense signal (**G–I**, yellow arrows) and corresponding DWI hyperintense signal (**J–L**, red arrows) both decreased. In a case of left TS and VL infarct (case 3), left temporal FLAIR hyperintensity decreases over time **(M–O)** as loss of flow-related enhancement resolves at the left TS on TOF MRV **(P–R)** and the left TS filling defect on T1 + C also resolves **(S–U)**. SSS, superior sagittal sinus; TOF MRV, time-of-flight MRI venography; TS, transverse sinus; VL, vein of Labbé.

During the subacute phase (days 6–15), methemoglobin release from red blood cell lysis causes thrombus to appear hyperintense on T1, T2, FLAIR, and T2^*^ sequences (2, 5). Susceptibility signal within thrombosed veins may improve at this stage. During the chronic phase (2–3 weeks), the thrombus becomes increasingly T1 isointense and T2 isointense to hyperintense and may appear heterogeneous with increasing visualization of flow voids as recanalization occurs (2, 21). During this period, the development of collateral circulation involving the cortical, epicranial, emissary, facial, and scalp veins may be seen. Enlarged collateral medullary veins may be perceived as prominent linear hypointense signal entering subependymal veins at right angles (7). Partial to complete recanalization of a thrombosed sinus may present a challenging diagnostic dilemma (21). In a retrospective analysis of 10 patients with chronic partially recanalized dural venous thrombosis, Leach et al. proposed three major components of chronic partially recanalized thrombosed veins: (1) enhancing organized thrombus, which demonstrates enhancement on T1 + C, no TOF MRV signal intensity, and diminished or lack of enhancement on C-MRV; (2) hypoenhancing thrombus, which shows no TOF MRV signal intensity and diminished enhancement on T1 + C images; and (3) recanalized channels, which demonstrate flow voids on T2 and T1 + C, flow-intensity related signal on TOF MRV, and continuous enhancement on C-MRV (21). Complete recanalization may occur more frequently in SSS and StS thrombosis than in TS or SS thrombosis (5). Table 4 summarizes the temporal evolution of the MR appearance of CVT. Figure 4 presents the images depicting the spectrum of MR findings seen in the temporal evolution of CVT.

**Table 4:** Summary of the classic MRI findings in the temporal evolution of CVT (2, 5, 6).

**Chronicity**	**T1**	**T2**	**FLAIR**	**GRE/SWI**	**T1 + C**
Acute (days 1–5)	Isointense	Very hypointense	Hypointense	Hypointense	Empty delta sign
Late acute	Mixed isointense, mildly hyperintense	Iso to mildly hyperintense	Iso to mildly hyperintense	Hypointense	Empty delta sign
Subacute (days 6–15)	Hyperintense	Hyperintense	Hyperintense	Heterogeneous, may have residual hypointensity	Enhancing dura surrounding hyperintense sinus
Chronic (2–3 weeks)	Isointense	Moderately hyperintense to isointense	Moderately hyperintense to isointense	Less hypointense	Enhancing dura ± collaterals (medullary veins)

*Chronicity refers to time from onset of CVT*.

### Imaging of CVI/CVT vs. AIS

CVI may cross arterial distributions on imaging and manifest as subcortical and periventricular white matter edema with cortical sparing, which may be accompanied by parenchymal hemorrhages (11, 15). While CBF to brain tissue is decreased in areas of arterial and venous occlusion, CBV is decreased in AIS but is normal or increased in CVI secondary to venous congestion (2, 15). On MRI, hyperintense signal abnormality on DWI with corresponding ADC hypointensity is convincing for AIS, whereas DWI signal abnormality may or may not be seen with CVT (1, 5). Favrole et al. reported that the presence of DWI signal abnormality in CVT correlates with lower rates of recanalization and persistent venous occlusion at 3 months after onset of treatment (19).

### Role of Advanced Imaging in CVT/CVI and Comparison With AIS

Current literature detailing the significance of arterial ASL and MRP in the imaging diagnosis of CVT is sparse. Both ASL and MRP are useful in characterizing CBF, and thus, vascular reserve in AIS has been incorporated into the routine protocol for AIS imaging at our institution. ASL perfusion imaging can quantify regional CBF *via* magnetic labeling of blood flowing into the brain (22); thus, decreased ASL signal within areas of cerebral infarct can be expected. MRP involves tracking an intravenous bolus of gadolinium contrast while rapidly imaging the brain to generate a signal intensity–time curve from which parameters such as relative cerebral blood flow (rCBF), relative cerebral blood volume (rCBV), and mean transit time (MTT) are derived. In AIS, areas of infarct exhibit increased MTT and decreased rCBF and rCBV, whereas the penumbra, an ischemic but potentially salvageable region surrounding the core infarct, manifests as decreased rCBF but normal or increased rCBV secondary to autoregulation (23).

The concept of the penumbra in AIS parallels the potentially reversible parenchymal injury in CVT before ischemic brain tissue from venous congestion progresses to definite infarction. As with AIS, perfusion changes can also be seen in CVT and CVI but are less well-understood. For example, studies have shown that CBV in this setting has significant variability (24–26). Some have demonstrated decreased CBV on CT perfusion (CTP), especially when hemorrhage is present (22, 27), whereas other studies have reported increased CBV in such cases (24). CBF, similar to its arterial counterpart, is typically decreased in areas of venous occlusion and infarct (15, 24, 27). Doege et al. concluded that perfusion imaging can also be useful in detecting reversible abnormalities (25). In six cases, they found that increased MTT when CBV was normal could represent reversible venous penumbra (25). In a prospective analysis of 20 patients with confirmed CVT who underwent CTP imaging before and after initiation of treatment for CVT, Gupta et al. agreed with Doege et al. in that there was a consistent increase in MTT, specifically, in the core and the periphery of the CVT-related lesion (25, 26). However, whereas Doege et al. had found that CBV was normal in patients with CVT, Gupta et al. noted a consistent and statistically significant decrease in CBV, which they attributed to a larger cohort of patients, post-processing algorithm differences, and the use of CTP data rather than MR perfusion data in their study (25, 26).

Rapid improvement in abnormal CBV and CBF values has been observed at time of follow-up imaging and with only partial recanalization of occluded dural sinuses after treatment (5, 24). Perfusion changes in CVT may reflect venous congestion and diminished CBF, which may be reversible prior to the development of permanent parenchymal damage or infarction (5, 6, 28).

In a retrospective analysis of 13 patients diagnosed with CVT by clinical and imaging criteria, Kang et al. observed increased ASL signal within thrombosed veins proximal to the area of thrombus, which they attributed to potentially excessive accumulation of labeled protons within the dural sinus proximal to the thrombus (27). They also noted that brain parenchymal hypoperfusion within the drainage territory of the thrombosed vein manifests as decreased signal and, thus, CBF on ASL images. Since ASL signal hyperintensity was noted in all of their 13 cases whereas the conventional findings of the empty delta sign and atypical distribution for an arterial territory were each found in approximately half of their cases, the authors postulated that the bright ASL signal might have higher sensitivity than the classic imaging findings of CVT (25). Furuya et al. reported two cases of CVT in which the brain parenchyma in the drainage territory of the thrombosed dural sinus initially demonstrated low ASL signal intensity, which improves soon after treatment but prior to the development of venous collaterals observed on MR angiography (MRA) (22). Their observations suggest that serial ASL imaging may predict an early response to treatment; since collateral development was not visualized on MRV, the authors suggest that improvements in ASL soon after initiation of CVT treatment may be accounted for by microcirculatory collateral development and improvement in perfusion of the capillary bed (22).

DWI hyperintense signal with corresponding ADC hypointense signal indicates restriction of free proton diffusion within areas of cytotoxic edema in the setting of AIS and is a well-established characteristic of the core infarct (19, 23). However, abnormal DWI signal in CVT is often variable and may be reversible (2, 19). In a study comparing DWI and ADC signal in the initial and follow-up imaging of 28 patients with CVT, Favrole et al. observed that when present, restricted diffusion was consistent with other findings of CVT on T1 and T2 FLAIR sequences (19). DWI and corresponding ADC signal abnormality were seen in 41% of patients with abnormal T2 signal intensity lesions associated with CVT with the exception of hemorrhagic lesions (19). Additionally, clinical symptoms persisted longer, and recanalization was less frequent in patients with diffusion restriction in the thrombus, leading to the postulation that DWI hyperintensity may be of value in predicting the risk of persistent venous occlusion upon follow-up imaging 3 months after the initial imaging diagnosis of CVT was obtained (19). In a retrospective analysis of 44 consecutive patients with CVT parenchymal lesions, Arnoux et al. found that even when DWI signal abnormality was present, corresponding ADC hypointensity was rare (9). DWI has low sensitivity for acute thrombosis but is helpful in the confirmation of parenchymal venous ischemia (7). The variable frequency with which diffusion restriction is seen in CVT as compared with AIS corroborates the more ischemic and thus potentially reversible nature of parenchymal injury in CVT as opposed to the irreversible cell death with tissue infarction in AIS.

## Case Presentations

### Case 1

**Figure 5:**
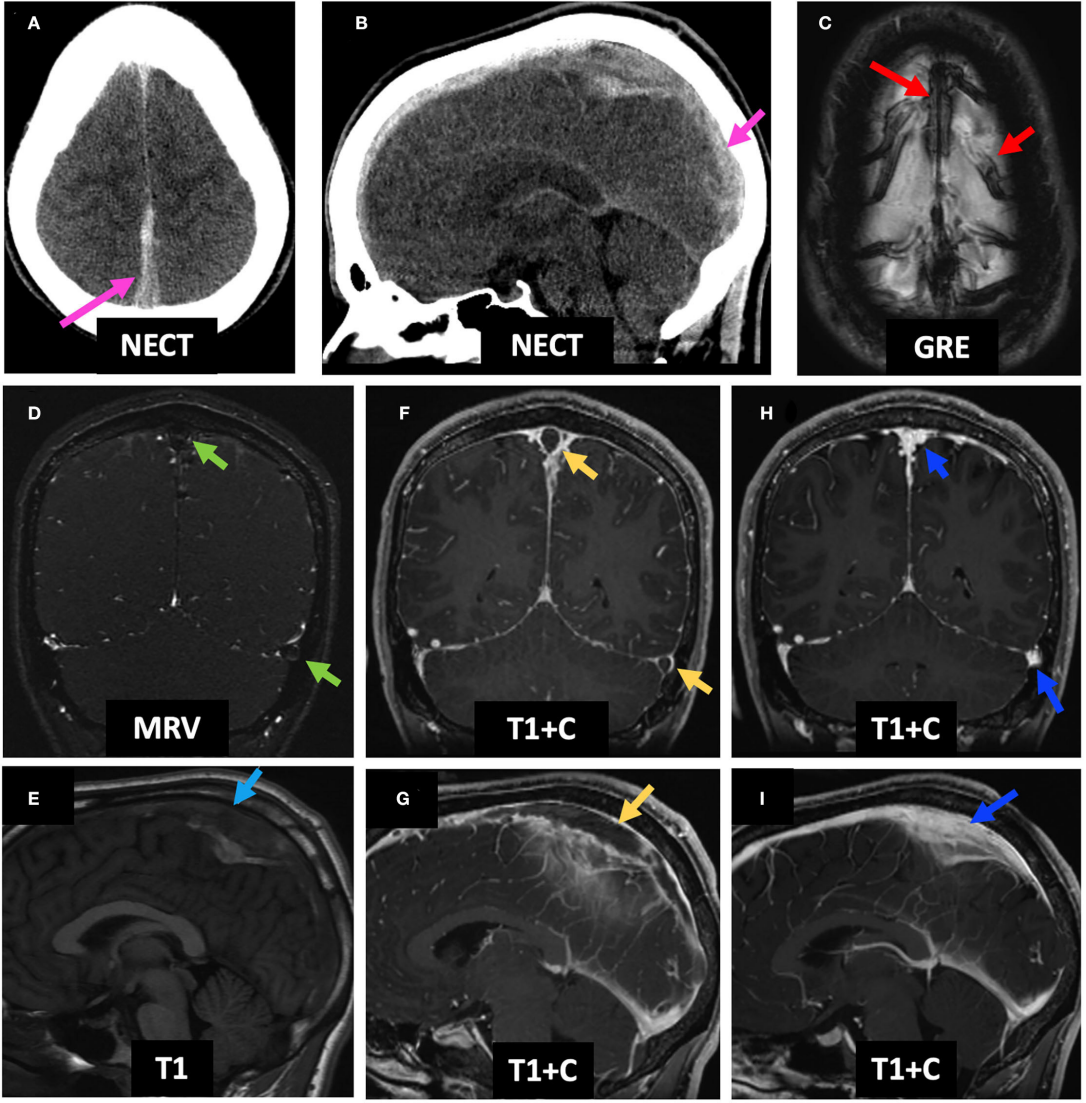
Case 1: An 18-year-old male who underwent a lumbar puncture 1 week prior to presentation was found to have worsening headache, dysarthria, and right hemiparesis with papilledema on exam. Initial NECT demonstrates hyperdensity of the SSS (**A,B**, pink arrows). C-MRI demonstrates GRE+ in the SSS and adjacent cortical veins (**C**, red arrows), lack of enhancement on MRV (**D**, green arrows), an expanded and T1 isointense SSS (**E**, light blue arrow), and empty delta sign in the SSS and left TS (**F,G**, yellow arrows). He received anticoagulation for 6 months. Follow-up C-MRI 4 months post-initial presentation demonstrates resolution of the GRE hypointensity and filling defects within the SSS and left TS, consistent with recanalization **(H,I)**. LP, lumbar puncture; NECT, non-contrast-enhanced CT; SSS, superior sagittal sinus; C-MRI, contrast-enhanced MRI; MRV, MR venography; TS, transverse sinus.

An 18-year-old male with 4 months of intermittent vague neurologic symptoms for which he received a lumbar puncture 1 week prior to arrival presented to the emergency department (ED) with worsening headache, dysarthria, acute right-sided weakness, and papilledema. On initial NECT, hyperdensity within the SSS and adjacent cortical veins was noted (Figures 5A,B). Initial contrast-enhanced MRI (C-MRI) demonstrated susceptibility within the SSS and adjacent draining cortical veins (Figure 5C), lack of enhancement on MRV (Figure 5D), expansile T1 isointense thrombus in the SSS on pre-contrast T1-weighted images (Figure 5E), and intraluminal filling defects within the SSS (“empty delta sign”) and left TS that extend to the SS on T1 + C (Figures 5F,G). No associated intraparenchymal edema, hemorrhage, or infarct was seen. Two days after presentation to the ED, he was initiated on anticoagulation with enoxaparin and continued on warfarin for 6 months; he was also given acetazolamide for papilledema that was weaned over time. The follow-up MRI 3 months later demonstrated resolution of filling defects and recanalization of the SSS and left TS (Figures 5H,I).

### Case 2

**Figure 6:**
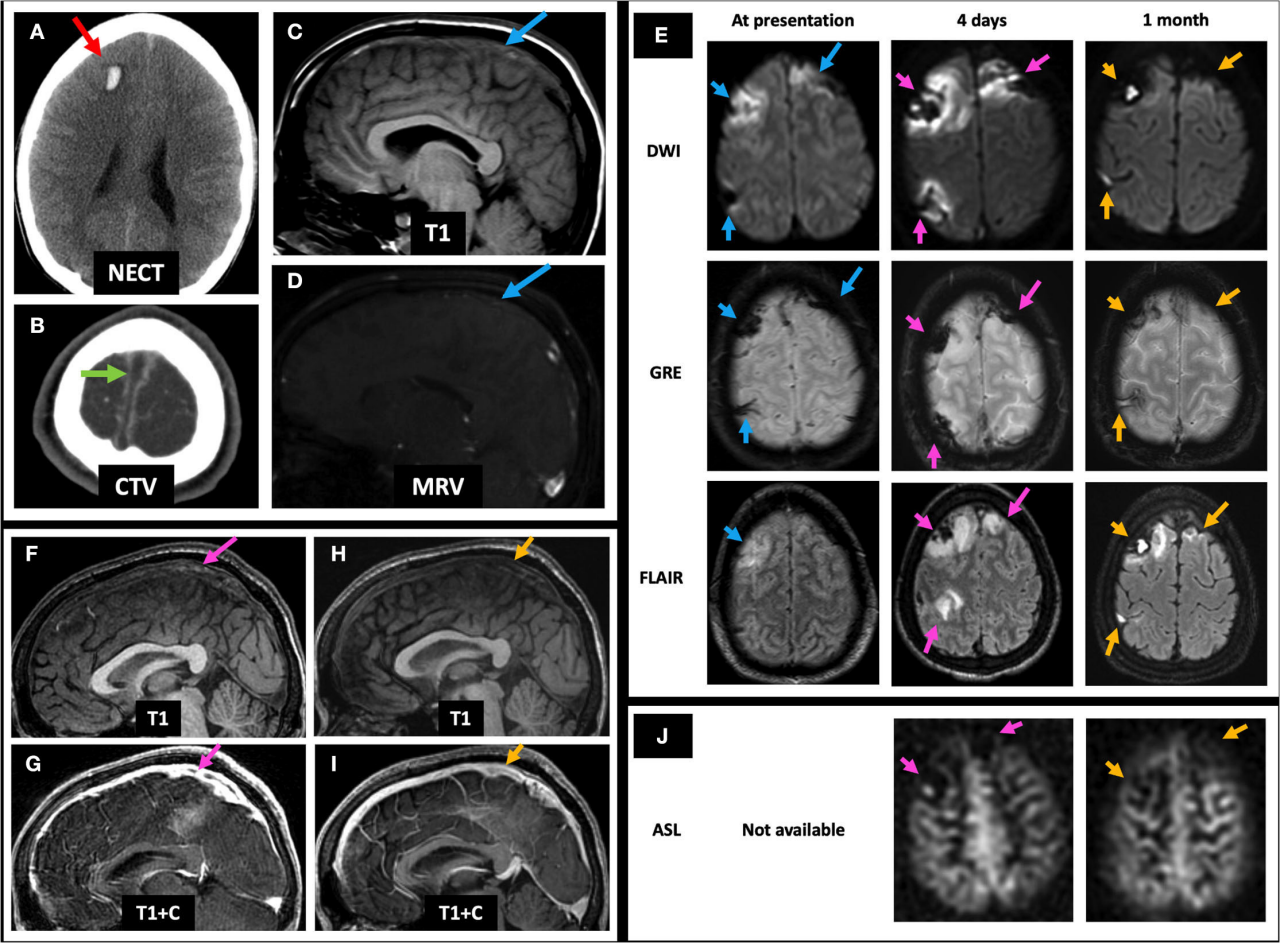
Case 2: A 23-year-old male with history of ulcerative colitis presented with headache, confusion, and agitation. Initial NECT showed bilateral frontal parasagittal hemorrhages (right parasagittal hemorrhage shown on **A**, red arrow). CTV showed a filling defect in the SSS (**B**, green arrow). Initial C-MRI/MRV showed subtle T1 isointense SSS thrombus (**C**, blue arrow) and no SSS flow-related enhancement (**D**, blue arrow). Mild bifrontal edema and hemorrhage are seen (**E**, left column, blue arrows). Four days later, the SSS thrombus appears mildly hyperintense (**F**, pink arrow) and demonstrates an empty delta sign (**G**, pink arrow), accompanied by slightly increased DWI and FLAIR signal abnormalities, as well as persistent GRE hypointensity (**E**, middle column, pink arrows). There is also associated right greater than left frontal hypoperfusion on ASL images (**J**, middle column). One month post-initial presentation, the SSS is again T1 isointense with resolution of filling defect (**H,I**, yellow arrows) and improvement in DWI, FLAIR, and GRE signal abnormalities (**E**, right column, yellow arrows), as well as improved but mild residual right greater than left frontal hypoperfusion on qualitative perfusion-weighted ASL images (**J**, right column). ASL, arterial spin labeling; CTV, CT venography; NECT, non-contrast-enhanced CT; SSS, superior sagittal sinus; C-MRI, contrast-enhanced MRI; MRV, MR venography; TS, transverse sinus.

A 23-year-old male with history of ulcerative colitis presented at an outside hospital 2 days prior to arrival with headache, confusion, and agitation. Initial NECT showed bilateral frontal parasagittal hemorrhages (with the right parasagittal hemorrhage shown on Figure 6A), and CTV demonstrated a filling defect in the SSS (Figure 6B), as well as within the right vein of Trolard, left TS, and left SS (not shown). Initial MRI showed T1 isointense thrombus in the SSS (Figure 6C) with lack of flow-related enhancement on TOF MRV (Figure 6D), as well as bilateral frontal hemorrhagic venous infarcts with associated DWI hyperintense, GRE hypointense, and FLAIR hyperintense signal abnormalities (Figure 6E, left column). On C-MRI 4 days later, the SSS thrombus appeared slightly hyperintense (Figure 6F), and the “empty delta sign” was appreciated on T1 + C (Figure 6G). The patient was initiated on enoxaparin with bridge to warfarin, which he continued for 6 months. On follow-up C-MRI/MRV 1 month later, complete recanalization of the SSS was seen with uninterrupted intrasinus enhancement and no residual filling defects (Figures 6H,I). Corresponding improvement in abnormal hypointense GRE signal and FLAIR and DWI hyperintense signal was seen from initial presentation to follow-up MRI 1 month later (Figure 6E, middle and right columns). Qualitative perfusion-weighted ASL images at 4 days follow-up demonstrate decreased perfusion at the right greater than left frontal lobes corresponding to the areas of hemorrhagic venous infarcts (Figure 6J, middle column), with improved but mild residual hypoperfusion on 1 month follow-up MRI (Figure 6J, right column). ASL images were not available for the MRI at presentation as the images were acquired at an outside institution.

### Case 3

**Figure 7:**
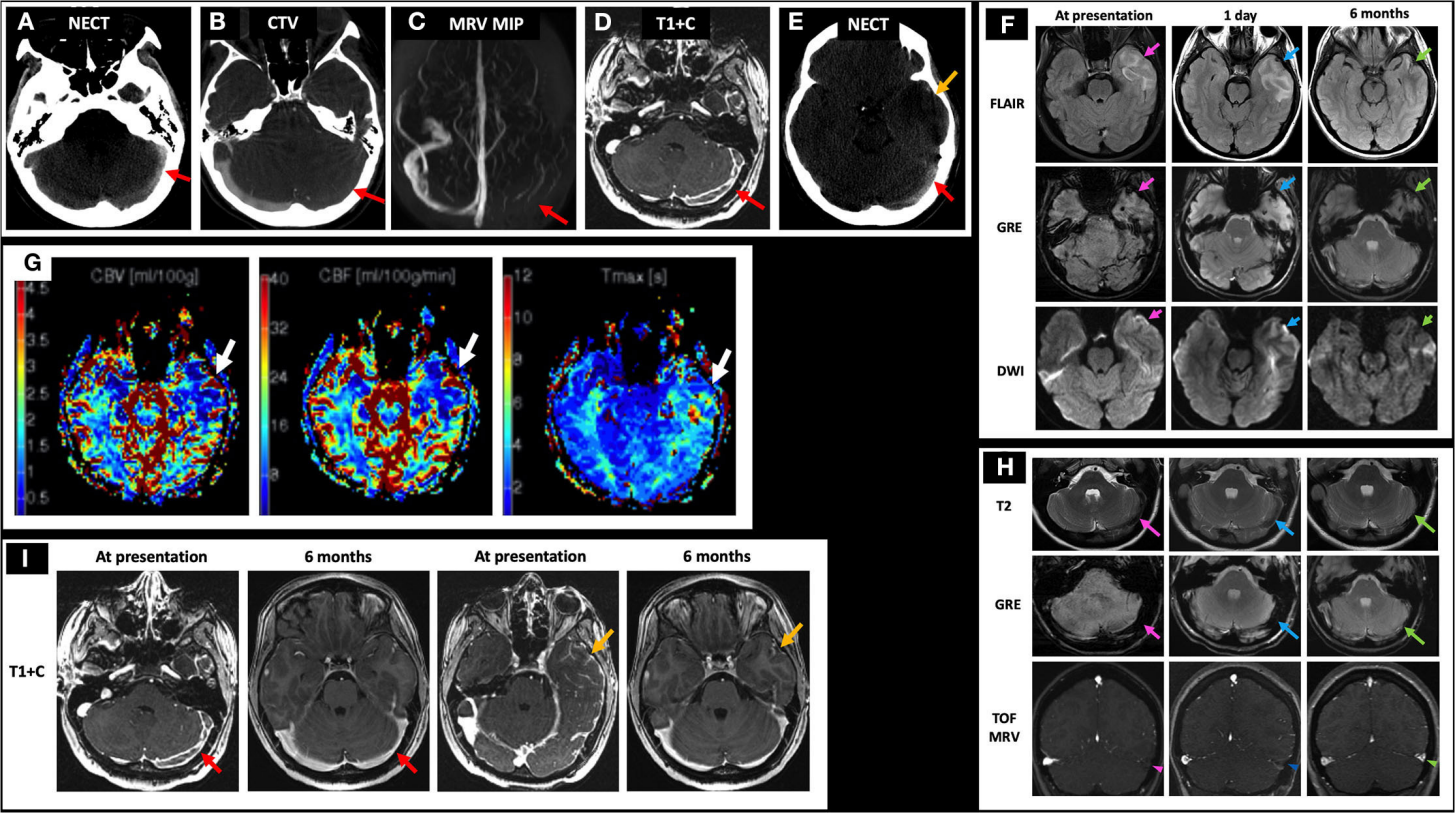
Case 3: A 19-year-old female with history of oral contraceptive use presented with 2 weeks of headache and witnessed seizures. Initial NECT showed hyperdense thrombus in left TS (**A**, red arrow), VL, and SS with associated filling defects on CTV and MRI/MRV (**B–D**, red arrows). One day after initiating anticoagulation, her NECT showed a left temporal hypodensity (**E**, yellow arrow) with MRI showing FLAIR hyperintensity, GRE hypointensity, and DWI hyperintensity (**F**, left column, pink arrows) but without corresponding ADC hypointensity (not shown). Bolus perfusion imaging showed decreased CBV and CBF and increased Tmax (**G**, white arrows). These findings represent vasogenic edema and petechial hemorrhage and thus ischemic but not infarcted tissue, consistent with CVT rather than CVI. Follow-up C-MRI/MRV 6 months later showed progression of the T2 hypointense thrombus to an isointense appearance (**H**, top row), improved susceptibility in the left TS (H, middle row), and resolved filling defects in the left TS on TOF MRV and C-MRV (**H**, bottom row; **I**, red arrows), as well as resolved left temporal gyriform enhancement (**I**, yellow arrows). On subsequent hypercoagulability work-up, she was found to have protein C deficiency. In **(H)**, the pink arrows point to the left TS thrombosis at presentation on T2, GRE, and TOF MRV, respectively (left column). The blue arrows point to the left TS thrombosis at 1 day follow-up on T2, GRE, and TOF MRV, respectively (middle column). The green arrows point to the left TS thrombosis at 6 months follow-up on T2, GRE, and TOF MRV, respectively (right column). CBF, cerebral blood flow; CBV, cerebral blood volume; CTV, CT venography; C-MRV, contrast-enhanced MRI venography; MRP, MRI perfusion imaging; TOF MRV, time-of-flight MRI venography; NECT, non-contrast CT; OCP, oral contraceptives; Tmax, time-to-maximum; TS, transverse sinus; VL, vein of Labbe; SS, sigmoid sinus.

A 19-year-old female with history of oral contraceptive use presented with 2 weeks of headaches and two witnessed seizures. Initial NECT showed hyperdense thrombus in the left TS, VL, and SS (not shown) and associated filling defects on CTV (Figures 7A,B). Initial MRI showed no flow-related enhancement in the left TS on TOF MRV and a corresponding filling defect on C-MRV (Figures 7C,D). One day after initiating anticoagulation, she was noted to have a left temporal lobe hypodensity on NECT (Figure 7E) with corresponding FLAIR hyperintense signal, diffusion restriction, and susceptibility artifact, concerning for CVI with petechial hemorrhage (Figure 7F, left and middle columns). On MRP of the left temporal lobe lesion, CBV and CBF were decreased, and time-to-maximum (Tmax) was increased (Figure 7G). On discharge, she continued warfarin and switched to dabigatran that she took for 6 months. Follow-up C-MRI/MRV 6 months later showed progression of T2 hypointense thrombus to an isointense appearance (Figure 7H, top row), improved susceptibility artifact in the left TS (Figure 7H, middle row), resolved filling defects in the left TS on TOF MRV and C-MRV (Figure 7H, bottom row; Figure 7I, red arrows), and resolved left temporal gyriform enhancement likely related to vascular congestion and BBB breakdown (Figure 7I, yellow arrows). On subsequent hypercoagulability work-up, she was found to have protein C deficiency.

### Case 4

**Figure 8:**
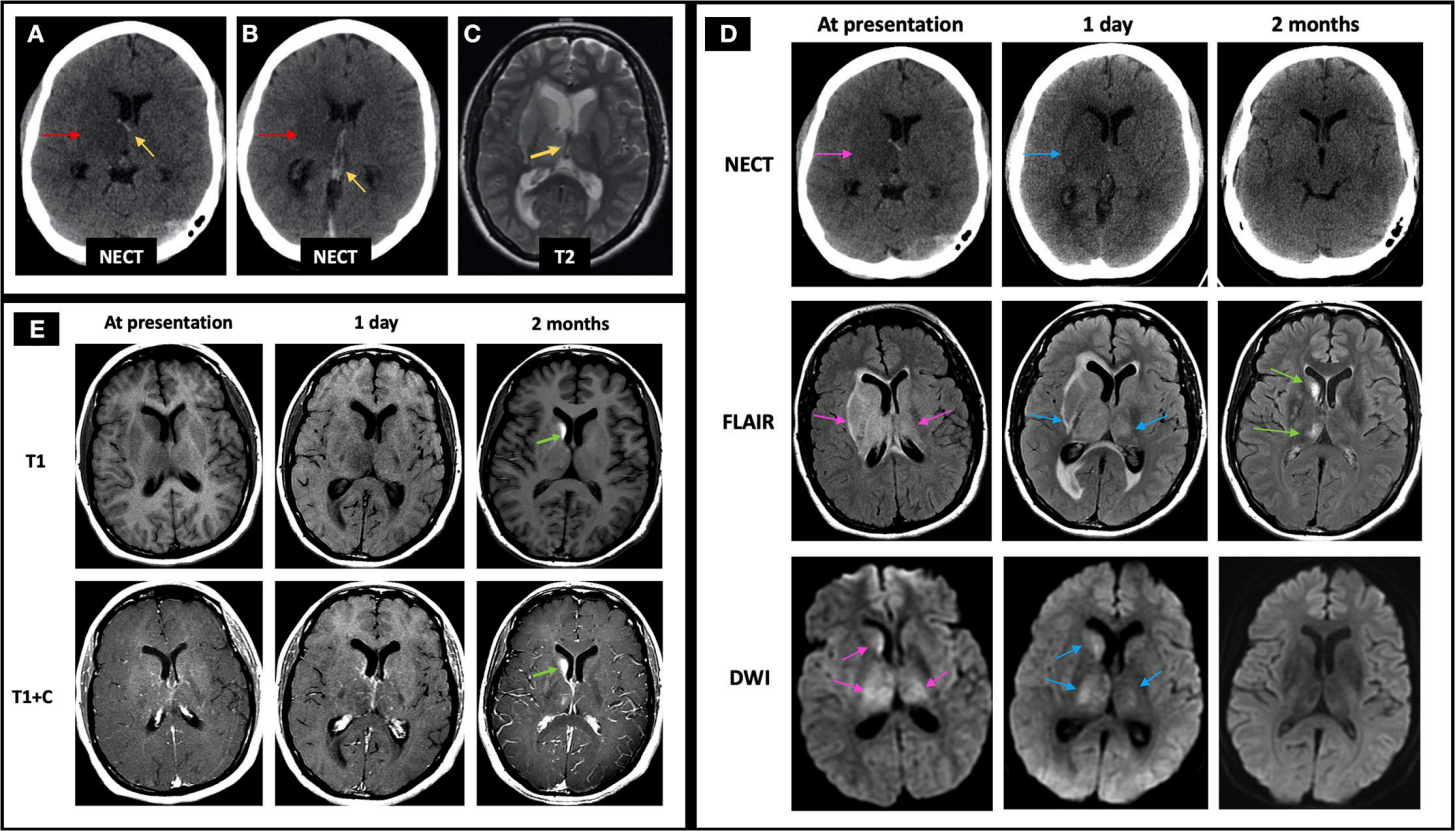
Case 4: A 43-year-old female presented with headaches, confusion, and left hemiparesis. Initial NECT showed curvilinear hyperdensity in the right greater than left ICVs (**A,B**, yellow arrows pointing to the ICVs, red arrows pointing to the adjacent edema) with associated T2 hypointensity on C-MRI (**C**, yellow arrow), suggestive of acute thrombus. FLAIR hyperintensity and diffusion restriction was seen at the right greater than left caudate, thalamus, and to a lesser degree the globus pallidus (**D**, left column, pink arrows) with mild increase in signal abnormalities 1 day later (**D**, middle column, blue arrows). At follow-up 2 months later, there is resolution of linear hyperdensity at the ICV (**D**, top row), as well as FLAIR and DWI hyperintensity at the right greater than left basal ganglia and thalamus (**D**, middle and bottom rows). Also, at 2 months follow-up imaging, the right caudate demonstrates encephalomalacia as evidenced by persistent FLAIR hyperintensity (**D**, middle row) and intrinsic T1 hyperintensity of the right caudate head on T1 pre-contrast images, compatible with pseudolaminar necrosis (**E**, green arrows). In **(D)**, the pink arrows point to the edema and related signal abnormality from ICV thrombosis at time of presentation on NECT, FLAIR, and DWI, respectively (left column). The blue arrows in **(D)** point to the same areas of signal abnormality at 1 day follow-up (middle column). The right column in D demonstrates near resolution of signal abnormality on NECT, FLAIR, and DWI at 2 months follow-up with green arrows pointing to mild residual FLAIR hyperintensity at the right basal ganglia and thalamus. C-MRI, contrast-enhanced MRI; ICV, internal cerebral veins; NECT, non-contrast CT.

A 43-year-old female presented with headaches, confusion, and left hemiparesis. Initial NECT shows curvilinear hyperdensity in the bilateral ICVs with right greater than left basal ganglia and thalamic edema (Figures 8A,B). Initial MRI demonstrated susceptibility artifact and lack of normal flow voids in the bilateral ICV (Figure 8C), VoG, and StS (not shown); right greater than left basal ganglia and thalamic FLAIR hyperintensity with dilated temporal horns with periventricular FLAIR signal abnormality suggestive of obstructive hydrocephalus (Figure 8D, middle row); and diffusion restriction consistent with CVI (Figure 8D, bottom row). Patient was initiated on heparin with bridge to warfarin and continued on dabigatran for 6 months. Follow-up MRI 2 months later revealed recanalization of the thrombosed deep cerebral veins with resolution of linear hyperdensity at the ICV on NECT (Figure 8D, top row), improvement in right greater than left basal ganglia and thalamic FLAIR hyperintensity (Figure 8D, middle row), and resolution of DWI hyperintensity (Figure 8D, bottom row). Additionally, right caudate encephalomalacia with intrinsic T1 hyperintensity was seen on 6 months follow-up (Figure 8E), consistent with pseudolaminar necrosis. She continued to have residual headaches on follow-up visits.

### Case 5

**Figure 9:**
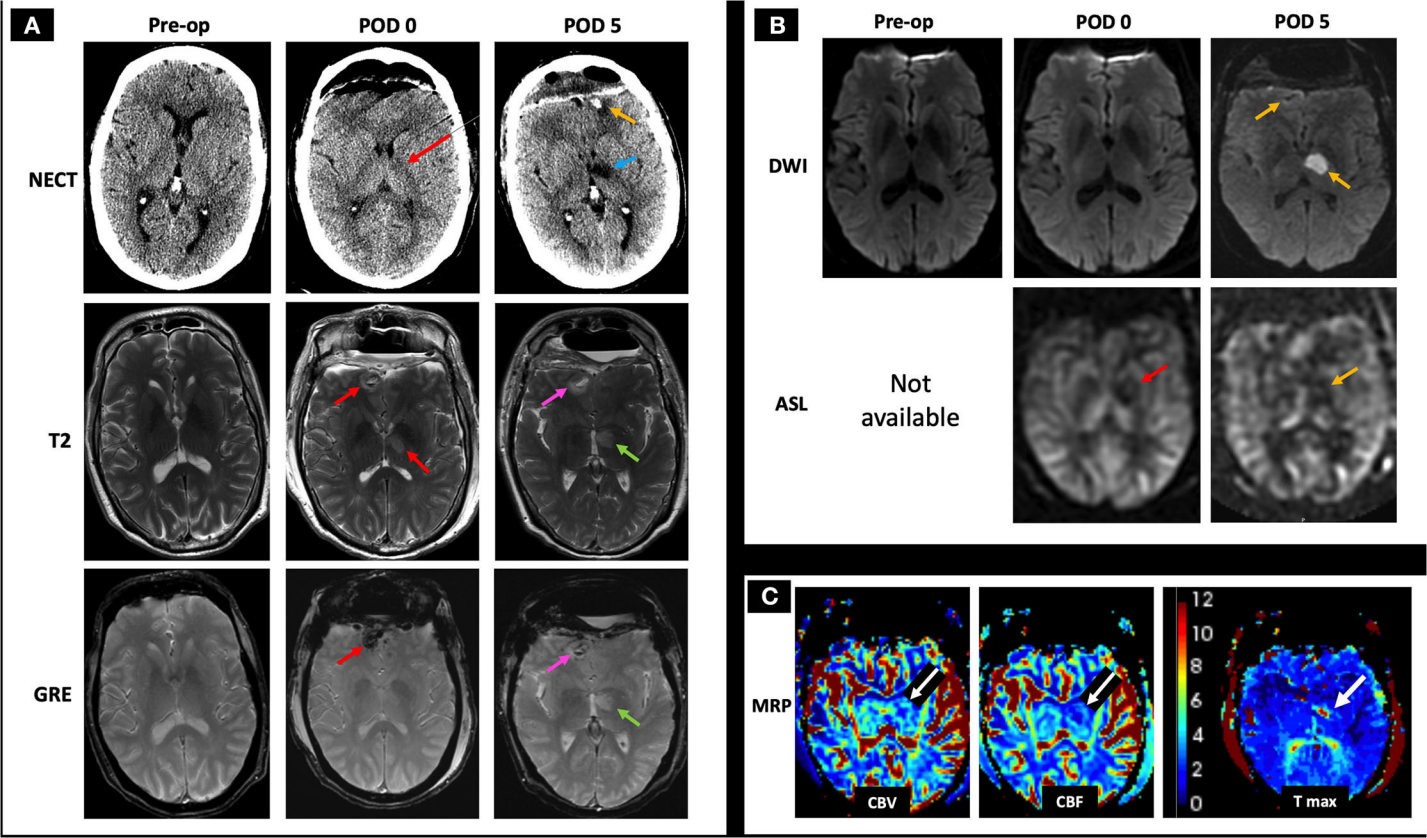
Case 5: A 57-year-old male with history of pituitary macroadenoma status post subtotal resection with subsequent bilateral frontal craniotomy for residual tumor removal 1 week prior to presentation developed acute altered mental status in the immediate postoperative period. The normal preoperative NECT, T2, GRE, and DWI images are included for comparison (**A**, left column; **B**, left column). Immediate postoperative (POD 0) NECT shows pneumocephalus with subtle hypodensity of the thalamus and posterior limb of left internal capsule (**A**, middle image in the top row, red arrow), and immediate postoperative MRI shows small bifrontal T2 hyperintense lesions with mild surrounding edema and associated GRE hypointensity (**A**, middle column, red arrows), as well as decreased ASL signal on qualitative perfusion-weighted ASL images without abnormal DWI signal (**B**, middle column, red arrow indicating hypoperfusion on ASL image), suspicious for hemorrhagic CVT. On follow-up 4 days later (POD 5), NECT showed a more conspicuous left thalamic hypodensity (**A**, right column, blue arrow) in addition to previously seen bifrontal hemorrhages (shown at the left frontal pole with a yellow arrow), concerning for infarcts. C-MRI showed T2 hyperintense signal and GRE hypointensity associated with the bifrontal hemorrhagic infarcts (**A**, right column, pink arrows point to the right frontal hemorrhage) and left thalamic infarct (**A**, right column, green arrows). Follow-up MRI (POD 5) showed diffusion restriction and decreased ASL signal at the left thalamus (**B**, left column, yellow arrows) with associated decreased CBV and CBF as well as increased Tmax (**C**, white arrows), consistent with CVT. Given the history of prolonged clamp time across the frontal lobe and the non-arterial distribution of these lesions, the bilateral frontal parenchymal hemorrhages are suggestive of iatrogenic CVT. AMS, altered mental status; CBF, cerebral blood flow; CBV, cerebral blood volume; C-MRI, contrast-enhanced MRI; ICV, internal cerebral veins; NECT, non-contrast CT; Tmax, time-to-maximum.

A 57-year-old male with pituitary macroadenoma status post transsphenoidal resection and bifrontal craniotomy 1 week later for residual tumor removal, onset postoperatively with acute altered mental status. The normal preoperative NECT, T2, GRE, and DWI images are included for comparison (Figure 9A, left column; Figure 9B, left column). Immediate postoperative NECT demonstrates postoperative pneumocephalus and subtle hypodensity of the left posterior limb of the internal capsule and thalamus (Figure 9A, middle image of top row), and immediate postoperative MRI demonstrates bifrontal T2 hyperintense lesions (right frontal lesion shown) with susceptibility (Figure 9A, middle column). Follow-up NECT 4 days later shows the development of bifrontal hemorrhagic venous infarcts thought to be due to prolonged clamp time across the frontal lobe and a more conspicuous left thalamic hypodensity (Figure 9A, top row). Follow-up MRI 4 days later shows diffusion restriction in the left thalamus and decreased ASL signal on qualitative perfusion-weighted ASL images (Figure 9B, right column) with corresponding decrease in CBV and CBF and increased Tmax (Figure 9C). In this postoperative case, the bifrontal parenchymal hemorrhages and left thalamic lesion show diffusion restriction not in an arterial distribution and were deemed CVI, likely iatrogenic in etiology.

### Case 6

**Figure 10:**
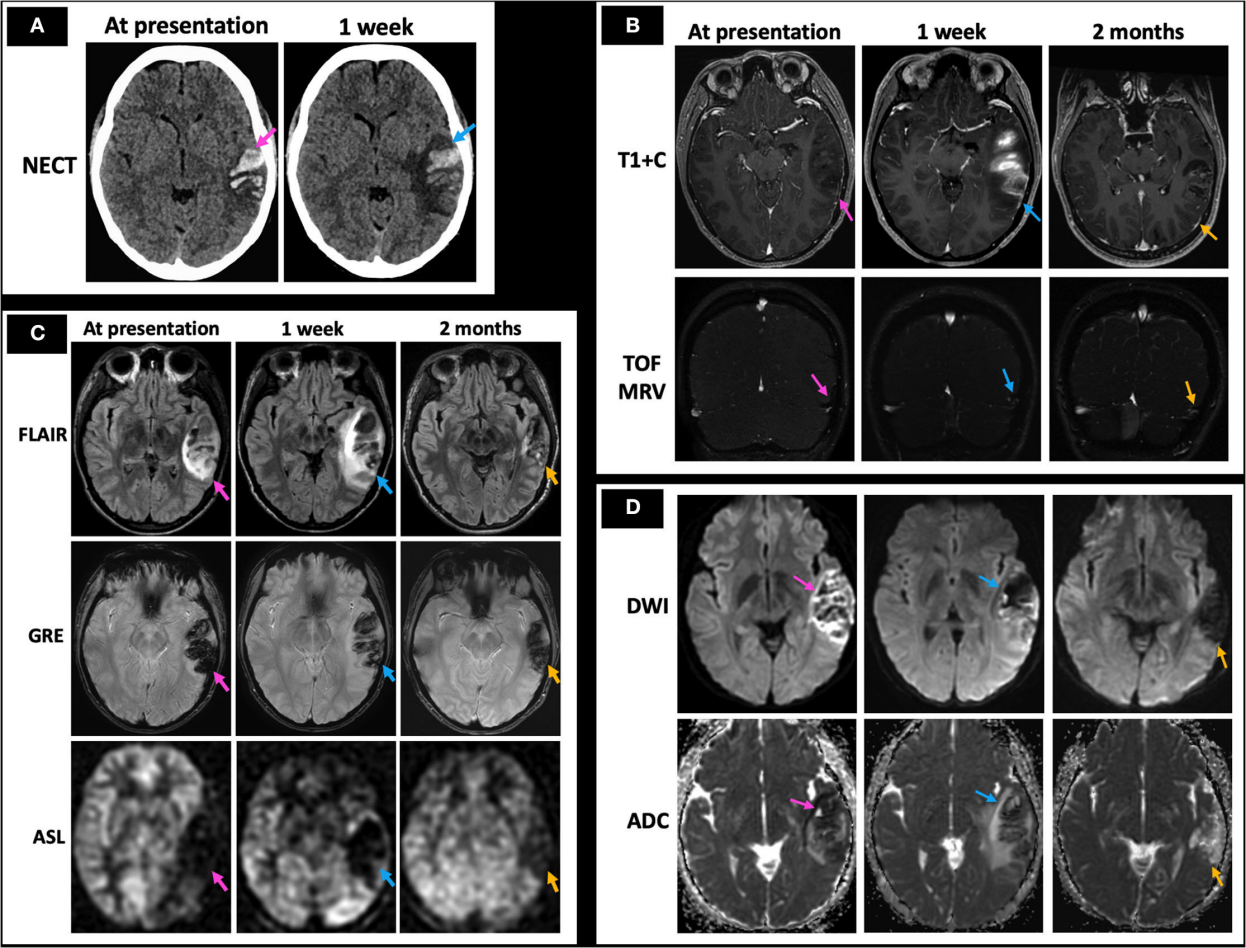
Case 6: A 26-year-old female with history of protein C deficiency, antithrombin III deficiency, and oral contraceptive use presented with difficulty reading and concentrating, as well as a left temporal headache. She was found to have non-fluent expressive and receptive aphasia on physical exam. Her initial NECT showed an acute left temporal hemorrhage (**A**, left image) with MRI demonstrating left vein of Labbé filling defects on T1 + C and TOF MRV (**B**, left column), consistent with left temporal hemorrhage secondary to a left vein of Labbé thrombosis. MRI at presentation also demonstrated surrounding FLAIR hyperintensity consistent with edema, prominent GRE hypointensity consistent with blood products, and hypoperfusion on qualitative perfusion-weighted ASL images (**C**, left column). She presented a week later with new right upper and lower extremity numbness, with her NECT demonstrating stable extent of the left temporal hemorrhage with increased surrounding edema (**A**, right image). There was also increased FLAIR hyperintensity consistent with increased edema, decreasing GRE hypointensity consistent with evolving blood products, and persistent hypoperfusion on ASL images (**C**, middle column) with a partially recanalized left vein of Labbé (**B**, middle column) and improving diffusion restriction (**D**, middle column). At 2 months follow-up, minimal residual filling defect in the left vein of Labbé is seen (**B**, right column) with markedly decreased left temporal edema as evidenced by decreased FLAIR hyperintensity and locoregional mass effect, decreased GRE hypointensity/blood products, and improved hypoperfusion (**C**, right column) along with resolution of diffusion restriction (**D**, right column). In **(A–D)**, the pink arrows point to the imaging findings at presentation, blue arrows point to findings at 1 week, and yellow arrows point to findings at 2 months. NECT, non-contrast CT; T1 + C, post-contrast T1-weighted images; TOF MRV, time of flight MR venogram.

A 26-year-old female with history of protein C deficiency, antithrombin III deficiency, and oral contraceptive use presented with difficulty reading and concentrating, as well as a left temporal headache. She was found to have non-fluent expressive and receptive aphasia on physical exam. Her NECT at presentation demonstrated acute left temporal hemorrhage with mild surrounding edema (Figure 10A, left image). Her MRI at presentation demonstrated a filling defect within the VL (Figure 10B, top row, left image) and associated loss of flow-related enhancement on TOF MRV (Figure 10B, bottom row, left image), consistent with left temporal hemorrhage secondary to a left VL thrombosis. Her initial MRI also showed increased FLAIR hyperintensity, prominent GRE hypointensity consistent with blood products, and hypoperfusion on qualitative perfusion-weighted ASL images (Figure 10C, left column), as well as diffusion restriction at the periphery of the hemorrhage (Figure 10D, left column) associated with the left temporal hemorrhage. She was initiated on anticoagulation with enoxaparin while she was admitted in the hospital and discharged on apixaban. A week later, she presented again with new mild right upper extremity and right lower extremity numbness. NECT demonstrated a stable region of left temporal hemorrhage with slightly increased surrounding locoregional edema (Figure 10A, right image). Her MRI at 1 week of follow-up demonstrated increased opacification of the left VL with increased flow-related enhancement (Figure 10B, middle column). Mildly increased edema surrounding the left temporal hemorrhage, less GRE hypointensity, and similar hypoperfusion (Figure 10C, middle column), as well as resolved diffusion restriction at the periphery of the lesion (Figure 10D, middle column), were also seen. As her symptoms were thought to be related to expanding edema, she was kept overnight for observation and discharged in the morning. Upon 2 months of follow-up, her MRI demonstrated markedly decreased FLAIR hyperintensity, continued decrease in GRE hypointensity, and improved hypoperfusion (Figure 10C, right column) with minimal residual filling defect in the left VL (Figure 10B, right column) and resolution of diffusion restriction (Figure 10D, right column). She was advised to avoid all estrogen-containing oral contraceptive and to continue on apixaban for life-long anticoagulation, given occurrence of the CVT.

## Discussion

CVT disproportionately affects young adults and children with a favorable prognosis of up to 80% when treated promptly (4). Anticoagulation is the mainstay of therapy with chemical or mechanical thrombolysis as considerations in the setting of severe neurological decompensation. AIS and CVI differ in important ways that influence treatment and prognosis. AIS is more prevalent in the elderly and patients with cardiovascular disease risk factors, whereas CVT is more often seen in younger and more female patients given the association of pregnancy and the postpartum state with CVT and those in a hypercoagulable state.

The imaging diagnosis of CVT is challenging in its varied and somewhat non-specific findings, compounded by generous variation in normal venous anatomy. Despite intraparenchymal hemorrhage, edema, and mass effect, tissue injury in CVT is more reversible than that in AIS (5, 15). We have reviewed the classic imaging findings of CVT and CVI in conjunction with ASL and MRI perfusion imaging, which has, to the best of our knowledge, not been explored together in the literature to date. In doing so, we highlight the potential utility of bolus and non-contrast perfusion imaging in detection of CVT and CVI.

Current perfusion techniques are not without limitations. Bolus perfusion techniques require the selection of an arterial input function (AIF) and assume that the AIF is the sole blood supply of the region. The choice and measurement of AIF are a common source of error (23). For example, mis-selection, partial volume averaging, and variability in choice of AIF can lead to non-diagnostic or variable blood flow measurements (23). Additional technical pitfalls include volume of interest (VOI) selection, delays in bolus arrival such as can be seen with variable cardiac output, severe intracranial vascular narrowing or multiple intracranial emboli, or inadequate flow within the vessels in the circle of Willis (29). MRI perfusion techniques are limited by low spatial resolution, orientation of the vessel with respect to magnetic field direction, and mixing of tissue signal intensity with signal from the artery (23, 29). The lack of standardization of post-processing tools across vendors, institutions, and modalities, e.g., CT vs. MR, is another major limitation (23, 29). Variability among CTP imaging maps among commercial software and between processing by technologists with different levels of experience suggests caution in interpreting quantitative CTP results (23). ASL and DWI have low signal-to-noise ratio and are susceptible to motion artifact. The presence of hemorrhage, not infrequently seen in CVT and CVI, confounds the interpretation of CTP parametric maps. MRP and ASL techniques are also susceptible to the magnetic field inhomogeneity caused by blood products (30). Larger studies are needed to more definitively assess the role of MRP and ASL in relation to outcomes in CVT.

Despite the limitations of perfusion imaging, there is emerging evidence that CTP imaging may be helpful for prognostication of CVT as well as be practical in the emergent clinical setting due to its reproducibility and rapid scan times. Gupta et al. performed a prospective analysis of CTP imaging data from 20 patients with confirmed CVT before and after initiation of treatment for CVT (26). CBF was consistently decreased in the center and periphery of the CVT-related lesion and was the best indicator of good clinical outcome among the parameters of CBF, CBV, and MTT (26). They also proposed that although there may not be a discrete region of infarct and penumbra, a region of perfusion deficit with rCBF > 60.5%, rCBV > 75.5%, and rMTT <148.5% would be associated with good clinical outcome (26). There was also a statistically significant improvement in perfusion parameters after initiation of treatment for CVT for the same cohort (26). Finally, the authors highlighted the practicality of using CTP imaging in emergent clinical situations given its short acquisition time, cost, and patient comfort, as well as the secondary and complementary role of TOF MRV that can also be performed with short acquisition times (26).

## Conclusion

CVI is a potentially life-threatening condition that has a more favorable prognosis if diagnosed earlier but is often underdiagnosed. AIS and CVI differ in important ways, including imaging characteristics, treatment, and prognosis. The imaging diagnosis of CVI is challenging in its varied and somewhat non-specific findings, compounded by generous variation in normal venous anatomy. This review provides a case-based overview of the classic imaging findings of CVT and CVI, as well as an exploration of the potential utility of perfusion imaging.

## Author Contributions

AL and ET: manuscript writing, image acquisition, editing, and reviewing. VY: idea generation, manuscript writing, image acquisition, editing, and reviewing. All authors contributed to the article and approved the submitted version.

## Conflict of Interest

The authors declare that the research was conducted in the absence of any commercial or financial relationships that could be construed as a potential conflict of interest.

## Publisher's Note

All claims expressed in this article are solely those of the authors and do not necessarily represent those of their affiliated organizations, or those of the publisher, the editors and the reviewers. Any product that may be evaluated in this article, or claim that may be made by its manufacturer, is not guaranteed or endorsed by the publisher.
